# Neuronal aging: learning from *C. elegans*

**DOI:** 10.1186/1750-2187-8-14

**Published:** 2013-12-10

**Authors:** Chun-Hao Chen, Yen-Chih Chen, Hao-Ching Jiang, Chung-Kuan Chen, Chun-Liang Pan

**Affiliations:** 1 Institute of Molecular Medicine, College of Medicine, National Taiwan University, No. 7, Chung-Shan South Rd, Taipei 100, Taiwan

**Keywords:** *C. elegans*, Aging, Neurons, Neural activity, Insulin signaling

## Abstract

The heterogeneity and multigenetic nature of nervous system aging make modeling of it a formidable task in mammalian species. The powerful genetics, simple anatomy and short life span of the nematode *Caenorhabditis elegans* offer unique advantages in unraveling the molecular genetic network that regulates the integrity of neuronal structures and functions during aging. In this review, we first summarize recent breakthroughs in the morphological and functional characterization of *C. elegans* neuronal aging. Age-associated morphological changes include age-dependent neurite branching, axon beading or swelling, axon defasciculation, progressive distortion of the neuronal soma, and early decline in presynaptic release function. We then discuss genetic pathways that modulate the speed of neuronal aging concordant with alteration in life span, such as insulin signaling, as well as cell-autonomous factors that promote neuronal integrity during senescence, including membrane activity and JNK/MAPK signaling. As a robust genetic model for aging, insights from *C. elegans* neuronal aging studies will contribute to our mechanistic understanding of human brain aging.

## Background

Aging is the common path that all organisms take to reach the ultimate end of a life time’s journey. For vertebrates that have an average life span of several decades, the entire process of aging can take as long as 20 years. For *Caenorhabditis elegans* (roundworm) or *Drosophila* (fruitfly) that live for 2-3 weeks or 2-3 months, respectively, aging occurs on a much smaller time scale of days to weeks. In spite of these differences in life span, however, genes or signaling pathways that influence aging of *C. elegans* or the replicative span of *Saccharomyces cerevisiae* (baker’s yeast) have been shown to be critical for longevity control in fly and in mice [1,2]. It seems that these common genetic pathways have adapted to the vastly diverse cellular and organismal contexts in different species to modulate the speed of aging appropriate to the time scale of their respective life spans.

In humans, aging of the nervous system compromises behavioral and cognitive functions, and predisposes to neurodegenerative diseases [3-6]. Tremendous efforts in recent years have been invested in the development of animal models for neurodegenerative diseases, such as Alzheimer disease (AD) and Parkinson’s disease (PD). Modeling human brain aging *per se*, however, receives relatively little attention. This dichotomy partly arises from the fact that mutations in single genes are associated with some forms of familial AD or PD, whereas few such mutations, if any, had been discovered to alter neuronal aging. Moreover, neuronal aging, similar to aging in other organs, is likely to be regulated by extensive interactions between genetic networks, signaling pathways and cellular metabolic responses, rather than a few genes. Any successful modeling of neuronal aging would therefore require an *in vivo* system that allows for extensive examination of multiple genetic pathways within the animal’s life span. Clearly, the powerful genetics and short life span (compared to the mice) of invertebrates such as worms and fly perfectly serve this need. The question is: do worm or fly neurons age in the same way as neurons in the human brain do?

### Aging of the *C. elegans* nervous system

#### Morphological aging of the neuron

Of the 959 somatic cells in an adult hermaphrodite *C. elegans*, 302 are neurons, making them the single largest group of postmitotic cells in this organism [7-10]. There are several major differences that distinguish *C. elegans* neurons from their mammalian counterparts. First, *C. elegans* neurons are small (5-10 μm of their soma size), and most of them do not have accompanying glial cells [10]. Sensory neurons of the amphid and phasmid, which are *C. elegans* sensory organs in the head and in the tail, respectively, are associated with glial cells but do not have myelination around their processes [10]. Second, they lack voltage-gated sodium channels, and whether *C. elegans* neurons generate typical action potentials is still an issue of active debate [11,12]. Third, most *C. elegans* neurons have simple morphology, do not have elaborate dendritic or axon arbors (except for the nociceptive FLP and PVD neurons [13-15]), and form synapses *en passant*[10]. And lastly, there is no neurogenesis in the adult *C. elegans*. Despite these differences, *C. elegans* neurons and mammalian neurons are remarkably similar in terms of functions and fundamental connectivity. In neural development research, *C. elegans* had been demonstrated as an extremely robust system to identify genetic pathways that regulate neuronal migration, axon guidance and synaptogenesis [16-18] (also nicely summarized in Wormbook: http://www.wormbook.org/toc_neurobiobehavior.html). Tiling and self avoidance, which are two mechanisms that ensure maximal, non-overlapping coverage of the sensory field between different neurons or within the same neuron, respectively, could be found in both mammalian and the *C. elegans* nervous systems [19,20]. Molecules critical for synaptic plasticity in the mammalian neurons, such as neuroligins, also regulate the function of the *C. elegans* synapses [21]. *C. elegans* has many sensory modalities common to human, such as olfaction, taste, thermosensation and mechanosensation; they sense oxygen and avoid noxious heat or mechanical stimuli (reviewed in Wormbook: http://www.wormbook.org/toc_neurobiobehavior.html). In all these sensory modalities, *C. elegans* displays remarkable behavioral plasticity that is akin to mammalian learning and memory [22].

In contrast to its robustness in developmental neuroscience, the potential usefulness of the *C. elegans* nervous system as a model for mammalian brain aging had been in great controversy until recently. In earlier studies that described age-dependent morphological changes in *C. elegans* tissues, no evidence was found for age-dependent structural deterioration in neurons, including well-preserved neuronal numbers, normal neurite placement, and grossly intact synapses and nuclear envelopes [23,24]. These descriptions posed a remarkable contrast to morphological aging in other somatic cells, where disorganization of myofibrils and cuticular collagen, emergence of cavities and alteration in nuclear architecture were evident in old animals [23,25]. It was difficult to reconcile the preservation of neuronal structures with the obvious age-dependent decline in pharyngeal muscle pumping, locomotion and various sensory behaviors [26-29], although some of the observed deterioration in motor responses could be a consequence of muscle degeneration.

**Figure 1:**
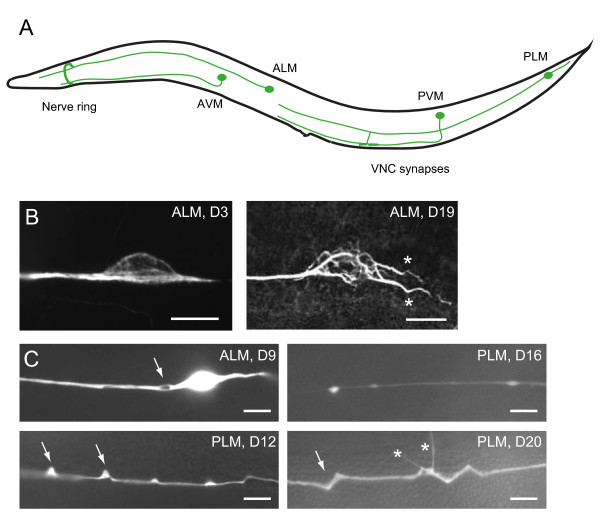
**Age-dependent defects of *C. elegans *touch receptor neurons. (A)** Schematic diagram of *C. elegans* touch receptor neurons, lateral view. For simplicity, only one of the bilateral ALM and PLM neurons was shown. VNC, ventral nerve cord. **(B)** Immunofluorescence of acetylated microtubules in the soma of young and old ALM neurons. Compared to the young neuron, the old ALM neuron showed aberrant sprouting from the soma (asterisks) and marked disorganization of microtubules. **(C)** Age-dependent axon defects in the touch neurons. ALM or PLM neurons were visualized in live animals with GFP expressed from the touch neuron-specific *mec-4* promoter. Arrows mark bubble-like lesions (upper left, ALM), beading (upper right, PLM), blebbing (lower left, PLM) and wavy processes (lower right, PLM). Asterisks label neurite branching in the PLM.

To solve this discrepancy regarding *C. elegans* neuronal aging, several recent studies reexamined the nervous system in old animals and revealed age-dependent morphological changes in senescent worm neurons [30-32]. Described in greater detail later in this article, these age-dependent neuronal defects include misshapen neuronal soma (Figure 1B), ectopic neurite branching (Figure 1B, C), and axon beading (focal swelling) or bubble-like lesion in the neuronal processes (Figure 1C). These age-dependent neuronal defects did not appear until the animals had entered adulthood, and were most evident in post-reproductive animals. This temporal profile distinguishes these defects from the neuronal maintenance defects described by Oliver Hobert and colleagues, which could appear in larvae of the mutants [33,34]. Most of the characterization was performed for the touch mechanosensory neurons, taking advantage of their simple morphology and well-resolved developmental genetics, but similar findings were also found in dopaminergic neurons and GABAergic motor neurons [30-32]. In the ventral nerve cord, the major longitudinal axon bundles that run most of the length of the animals, cholinergic axons displayed age-dependent defasciculation and became less compact [30]. Disorganized microtubule arrays were observed in touch neurons with misshapen soma [30]. Mitochondria could be found at the small axon swellings or at the sites of ectopic branching in the process of the touch neurons [32]. A reduction in the density of synaptic vesicles was also documented at the synapses in the nerve ring, the major neuropil of *C. elegans*[32]. While these reports confirmed the conclusion made by earlier studies that there were no neuronal loss or axon placement defects in aging *C. elegans*, they clearly demonstrated that age-dependent deterioration of neuronal structures does occur at the subcellular level.

Are these age-dependent neuronal defects similar to those found in the nervous system of other species? Dystrophic neurites characterized by abnormal sprouting and varicosity formation or axonal spheroids had been described in senescent human brain [35]. These morphological defects were reminiscent of the neurite branching or axon beading found in the *C. elegans* touch receptor and GABergic neurons [30-32]. Importantly, no significant neuronal loss is found in most areas of the aging mammalian brains, including those of the primates [3,4]. Age-dependent decline in synaptic organization were found in the neuromuscular junction of *Drosophila* and mouse, and at the rat cerebellar synapses [36-38]. Purkinje cells, which are the projection neurons in the cerebellum, in the aged rats showed decreased number of small dendritic spines and increased density of larger dendritic spines [38,39]. Defects in presynaptic structures and synaptic vesicle density were also documented in *C. elegans*[32]. Therefore, it seems that some of the fundamental phenomena in neuronal aging are conserved in *C. elegans* and in other species.

#### The cell biology of *C. elegans* neuronal aging

**Figure 2:**
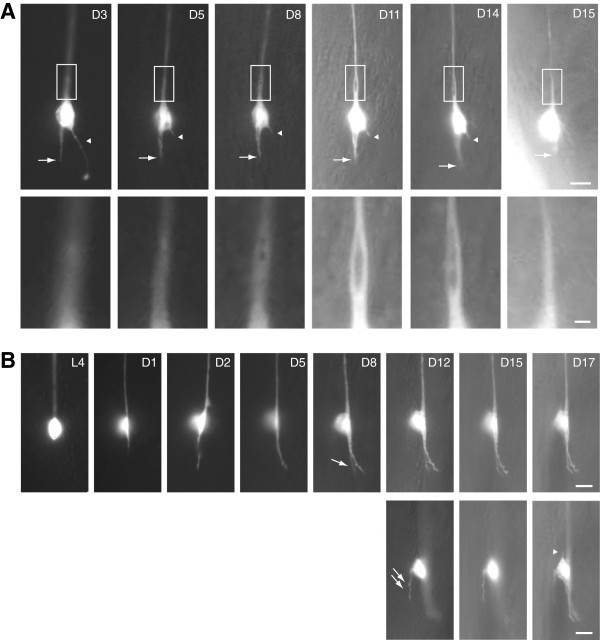
**Temporal evolution of neuronal defects during aging in *C. elegans*.** The same ALM neurons in the wild type were imaged at different time points over the animals’ lifespan; lateral view, anterior is up. Neurons were labeled by a touch cell-specific GFP reporter. Scale bar = 5 μm or 1 μm (A, insets). **(A)** The posterior process of the ALM neuron (arrow) remained static from D5 to D14 but retracted later. Arrowheads mark an ectopic sprouting from the soma, which was truncated between D3 and D5, and completely retracted on D15. Insets highlight the development of a bubble-like lesion in the proximal ALM process. The animal died on D16. **(B)** The ALM grew a posterior process on D1, which continued to lengthen between D1 and D5, and branched at D8 (arrow). An ectopic branch emerged from the dorsal side of the cell body at D12 (lower panel, double arrows). On D17, another short sprouting grew at the anterior aspect of the neuron (arrowhead). The three images of the right lower panel were taken from different focal planes. Images were originally published in the Proceedings of the National Academies of Sciences of the U.S.A. and reused with permission [30].

How morphological aging of the neuron occurs over time is a fundamental but largely unanswered question. Even with the advance in imaging technology, imaging the same neuron over the entire life span of individual animals remains a daunting feat in mammals. This is where the simple neuronal architecture, body transparency and short life span of *C. elegans* could reveal unprecedented novel insights into the evolution of age-dependent neuronal defects over the entire adulthood of the animal. By imaging the same touch neuron over the entire adulthood of the worm until its death, Pan et al. reported striking dynamic changes of neuronal sprouting during the course of aging (Figure 2) [30]. New sprouts could emerge from the neuronal soma as late as day 12, a relatively late time point in a wild-type *C. elegans* that lives two to three weeks on average [30]. The emergence of supernumerary sprouts likely represents a failure in cellular mechanisms that would normally prevent the formation of ectopic structures, rather than an enhanced ability to generate new branches in response to the wear-and-tear aging process, because the ability to regenerate the axon following axotomy was drastically reduced in adult *C. elegans*[40-43]. Retraction or splitting of neurite sprouts was also observed. Large bubble-like lesions formed by coalescence of smaller vesicle-like foci in the proximal touch neuron processes [30]. Although various axonal defects had been documented, such as beading, blebbing or bubble-like lesions, they could represent different stages of the same pathology in active progression. For example, impairment in axon transport may cause focal cytoplasmic swelling seen as beading, which may further progress to impede the transport of large organelles, such as mitochondria, and form small bubble-like lesions where the obstructed organelles excluded the cytoplasmic GFP signal [32]. On the other hand, large bubble-like lesions probably reflect the early phase of axon splitting, because the bubble could extend over long distance and was not labeled by organelle markers [30]. While these morphological characterizations did not identify the molecular mechanisms behind neuronal aging, they did provide a framework and context for future mechanistic studies.

#### Functional aging of the neurons

Age-dependent deterioration in neuronal morphology suggests that neuronal dysfunction contributes to motor and behavioral decline in old *C. elegans*. Cai and Sesti had shown that age-dependent oxidation of the potassium channel KVS-1 was responsible for the observed decline in the chemotactic behaviors of old worms, and expression of an oxidation-resistant KVS-1 in the chemosensory neurons conferred resistance to age-dependent chemotactic deficits [44]. While this observation strongly suggested that neuronal dysfunction occurs in old worms, a direct measurement of neuronal activity in aged animals had been lacking, owing to the technical difficulty of recording electrical activity from the relatively small *C. elegans* neurons. A breakthrough came when Liu et al. assayed the function of cholinergic and GABAergic motor synapses by directly recording their postsynaptic muscle cells [45]. While activation of the muscle membrane, measured as the postsynaptic current (PSC) in electrical recording, has contributions from both the presynaptic neuron (neurotransmitter release) and the postsynaptic muscle (density and function of the neurotransmitter receptors), the frequency of spontaneous PSC is an accurate indicator for presynaptic release function. Liu et al. observed a progressive decline in the release of neurotransmitters from the presynaptic terminal that began as early as day 7, which correlated with the decrease in locomotion and suggested age-dependent neuronal dysfunction [45]. Strikingly, function of the muscle, judged from the evoked PSCs and the contractibility induced by the cholinergic agonist levamisole, was preserved at the time when presynaptic functions started to deteriorate, and only began to show deficits between day 11 and day 15 [45]. Additional electrophysiological characterization by the authors led to the conclusion that middle-age motor neurons first developed defects in synaptic vesicle fusion, followed by deficits in neurotransmitter contents and synaptic vesicle priming or docking at the neuromuscular junction [45].

While an early decline in neurotransmitter release at motor synapses had been established in *C. elegans*, other dimensions of neuronal functions, such as membrane excitability and axon transport, had not been examined in the context of aging. For example, does the emergence of beading or bubble-like lesions in the touch neuron processes correlate with compromised cargo transport in these cells? Do aberrant neurite branching or wavy processes interfere with the generation or propagation of electrical activity along the neuronal membrane? Since many behavioral correlates of neuronal functions in *C. elegans* depend on locomotion responses that are heavily affected by muscle contractility, answers to these questions will require direct measurement of neuronal activity by electrophysiology or fluorescence-based methods. Several recent technological advances make it possible to begin to address these questions fundamental to neuronal aging. For example, FRET (förster/fluorescence resonance energy transfer) or GCaMP-based measurement of neuronal calcium transients in animals immobilized in a microfluidic device allows for assessment of neuronal activity in response to various sensory stimuli [46-50]. With the addition of optogenetic tools derived from the channelrhodopsin and related light-sensitive channels [51,52], it is becoming possible to address functional decline in the aging *C. elegans* nervous system, at single-cell level, in the context of intact neuronal circuits.

### Genetic control of *C. elegans* neuronal aging

#### Neuronal aging is influenced by longevity genes

Mutations that alter the worm life span had been shown to affect the speed of tissue aging in a manner similar to how these mutations influence longevity. For example, mutations in the FOXO3 transcription factor *daf-16* and the heat shock transcription factor *hsf-1* shortened life span and accelerated morphological aging of the head and the germline (Table 1) [1,2,25,53-55]. By contrast, mutations in *daf-2*, which encodes the insulin-like growth factor (IGF) receptor and inhibits *daf-16* functions, dramatically extended the life span and delayed tissue aging [1,2,53,56]. The impact of these longevity genes on the structural and functional aging of *C. elegans* neurons was also confirmed. In short-lived *daf-16* or *hsf-1* animals, neurons displayed more defects at earlier ages, and genetic analysis indicated that *daf-16* and *hsf-1* function in a common pathway [30-32]. In *daf-2* animals with a long life span, many of the structural defects were delayed or ameliorated, and the decline in presynaptic functions was dramatically reduced [30-32,45]. Mutations in *clk-1*, a demethoxyubiquinone hydroxylase required for mitochondrial respiration, extended life span and ameliorated excessive neurite branching in old animals [31].

**Table 1:** ***C. elegans *** **genes that regulate neuronal aging**

**Genes**	**Identity**	**Major function**
Genes that promote neuronal integrity during aging		
*daf-16*	FoxO transcription factor	Upregulation of stress resistance genes
*hsf-1*	Heat shock transcription factor	Upregulation of small heat shock protein expression
*mec-1*	EGF and Kunitz domain-containing ECM protein	Mechanosensory transduction, nerve attachment
*mec-2*	Stomatin-like	Mechanosensory transduction
*mec-4*	DEG/ENaC family sodium channel	Mechanosensory transduction
*mec-5*	Atypical collagen	Mechanosensory transduction, nerve attachment
*mec-6*	Paraoxonase	Mechanosensory transduction
*mec-9*	EGF and Kunitz domain-containing ECM protein	Mechanosensory transduction, nerve attachment
*mec-10*	DEG/ENaC family sodium channel	Mechanosensory transduction
*mec-12*	α-tubulin	Mechanosensory transduction, axon transport
*jkk-1*	JNK kinase	Activation of JNK; synaptic vesicle localization
*jnk-1*	c-Jun N-terminus kinase (JNK)	Heat and stress response; locomotion
*mek-1*	Mitogen activated protein kinase (MAPK) kinase/MKK7	Stress and starvation response
*lmn-1*	Laminin	Regulation of nuclear organization and function
*ptl-1*	Tau/microtubules-associated protein	Microtubule stabilization?
*unc-13*	Munc-13	Synaptic vesicle fusion
*unc-18*	Munc-18	Synaptic vesicle docking
Genes that accelerate neuronal aging		
*daf-2*	Insulin-like growth factor receptor	Larval development, dauer formation, etc
*dgk-1*	Diacylglycerol kinase	Inhibition of synapse transmission
*slo-1*	BK-type potassium channel	Muscle inactivation, neuronal excitability, etc

Longevity genes could cell-autonomously act in the neurons to influence cellular aging. Alternatively, they may impact neuronal aging by modulating life span at the organismal level, acting non-autonomously outside the nervous system. The findings by Pan et al. were consistent with this view, where expression of *daf-16* in the neurons failed to rescue premature neuronal aging of the *daf-16* mutant [30]. Since *daf-16* functions largely outside the nervous system to regulate *C. elegans* life span [1,2], this observation indicates that accelerated neuronal aging in the *daf-16* mutant is a consequence of shortened life span at the whole animal level. These findings are also consistent with a recent report that homeostasis of protein quality control, an important factor in cellular aging, in the *C. elegans* muscles could be regulated non-autonomously [57]. However, Tank et al. found that re-introduction of *daf-16* in the neurons of the *daf-16; daf-2* double mutant made the touch neurons look similar to the *daf-2* mutant touch neurons, with fewer age-dependent neurite branching compared to the wild type [31]. While this was a context slightly different from that in the study by Pan et al. and focusing only on neurite branching but not other types of neuronal aging defects, it seemed to suggest that at least for preventing ectopic branching, *daf-16* could act cell-autonomously in the neurons. It remains to be determined whether *daf-16* acts in more than one tissue to regulate cellular aging of the neurons.

It had been shown that behavioral decline in the mouse Aβ-based AD model was partially ameliorated by eliminating one copy of the IGF receptor. At the cellular level, compared to control AD mice, reactive astrogliosis (proliferation of astrocytes) was also reduced in *Igfr(+/-)* AD mice. While direct evidence that reduction of insulin signaling delays mammalian neuronal aging is pending, these observations support the idea that genetic mechanisms of aging are conserved in *C. elegans* and in mammals, and that manipulations that delay organismal aging can be promising disease-modifying therapeutics for human neurodegenerative disorders.

#### Cell-autonomous factors for neuronal aging

**Figure 3:**
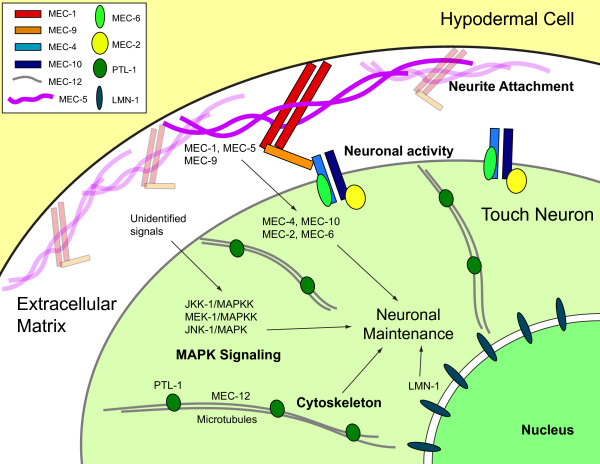
**Schematic model of genetic and signaling networks that regulate maintenance and aging in *C. elegans *touch neurons.** The touch neurons and their processes are ensheathed by the cytoplasmic extension of the neighboring hypodermal cell. Extracellular matrix containing the EGF- and Kunitz-domain proteins MEC-1 and MEC-9, and also atypical collagen MEC-5, was deposited between the touch neurons and the hypodermal cell. It is generally speculated that MEC-1, MEC-5 and MEC-9 tether the mechanosensory transduction channels, composed of MEC-2, MEC-4, MEC-6 and MEC-10, on the touch cell membrane and mechanically gate these channels. Although Tank et al. [31] had shown that components in the MAPK pathways, including JNK-1, JKK-1 and MEK-1, maintain touch neuron structures by inhibiting aberrant branching during aging, signals that activate these genes as well as their effectors or targets remain elusive. Genes that encode components of the microtubule cytoskeleton (MEC-12/α-tubulin and PTL-1/Tau) or the integral nuclear envelope protein (LMN-1/lamin) are also important for maintaining postmitotic neurons in *C. elegans*. For simplicity, this schematic diagram was generated in the form of the neuronal soma, but similar models could also apply to the process of the neuron.

Non-autonomous factors regulate the speed of aging across different tissues and serve to coordinate aging in individual tissues at the organismal level. By contrast, genes that act directly in the neurons impact neuronal aging by modulating cellular structures and functions (Figure 3). A set of *mec* (for *mechanosensitivity*) genes are specifically expressed in the touch neurons and essential for their activation by sensory input [58-60]. These include genes that encode DEG/ENaC-type sodium channels (*mec-4*, *mec-10*) [58,59,61], channel accessory proteins (*mec-2*, *mec-6*) [58,62], extracellular matrix proteins (*mec-1*, *mec-5*, *mec-9*) [58] and touch neuron-specific tubulins (*mec-7*, *mec-12*) [63,64]. Pan et al. found that in many of these *mec* mutants (*mec-7* was not tested), touch neurons showed premature neuronal aging due to a lack of sensory-evoked membrane activity [30]. When synaptic transmission was blocked, GABAergic neurons showed increased axon beading, suggesting that membrane activation by presynaptic input or the synaptic release function is necessary to prevent premature aging of the neuron [30]. Additional age-dependent pathology of wavy axon, axon blebbing and branching was observed in the *mec-1* and *mec-5* mutants, in which the normal attachment of the touch neurons to the neighboring hypodermal cells was disrupted [30]. Tank et al. found that the c-Jun terminal kinase *jnk-1* and its upstream kinases, *jkk-1* and *mek-1*, were required to prevent the formation of ectopic neurite branching during aging [31]. The *jnk-1*, *jkk-1* and *mek-1* mutants showed more neurite branching than the wild type, and the increase in supernumerary branches of the *jnk-1* mutant could be eliminated when *jnk-1* expression was restored to the neurons [31]. Recently, it was reported that PTL-1, the *C. elegans* homolog for the microtubules-associated protein Tau, was required for adult touch neuron maintenance [65-67]. The touch neurons in the *ptl-1* mutants showed more age-dependent defects than the wild type [67]. Microtubule disorganization occurred in the soma of senescent touch neurons [30], so it is possible that PTL-1 maintains normal neuronal structure during aging by regulating microtubule structures. Mutations in the integral nuclear envelope protein LMN-1/lamin triggered premature neuronal aging and markedly shortened life span [24,30]. In summary, genes that promote membrane activity, nerve attachment, stress response (the JNK/MAPK pathway) and the integrity of cytoskeleton and nuclear envelope are involved in maintaining postmitotic neurons against aging.

As described above, many mutations that alter the progression of neuronal aging also influence life span, raising the possibility that the observed changes in the speed of neuronal aging are merely a consequence of organismal aging. *jnk-1*, *ptl-1* and several *mec* mutant strains had shortened life span [30,31,67], and it remains to be determined whether the increase of age-dependent neuronal defects in these mutants is a consequence of premature aging at the organismal level. Several lines of evidence, however, indicate that neuronal aging and life span could be uncoupled. First, *mec-1* and *mec-12* animals showed comparable life span to that of the wild type, indicating that the observed increase in age-dependent neuronal defects of these two mutants was not secondary to life span reduction [30]. Second, mutations in *eat-2*, which encodes a nicotinic acetylcholine receptor (nAChR) subunit [68], increase the worm life span by reducing pharyngeal pumping and food intake; nevertheless, *eat-2* animals showed neuronal aging comparable to that in the wild type [30]. Clearly, cellular senescence could progress independent of organismal aging.

### Is there proteinopathy in *C. elegans* neurons?

One of the most important but poorly understood questions in modeling human brain aging with *C. elegans* is the involvement of abnormal protein aggregates, the so-called “proteinopathy” theory. In the normal aging brain of human, extracellular aggregates of β-amyloid peptides could be found in small quantity, with occasional intracellular Tau aggregates in the neurons [3-6,35]. The significance of these asymptomatic protein aggregates in human neuronal aging is a matter of much controversy. The *C. elegans* APL-1 is a homolog for human β-amyloid precursor protein [69,70]. Although neuronal APL-1 overexpression disrupted several behaviors such as olfactory and gustatory learning or touch habituation [71], whether or how neuronal structures and functions were impaired was undetermined, and documentation of APL-1 protein aggregation was not addressed. It is unknown either whether APL-1 aggregation is part of physiological aging in *C. elegans* neurons. As described above, mutations in the *C. elegans* Tau gene *ptl-1* resulted in touch insensitivity, premature touch neuron aging and shortened life span [67]. While introducing the human Tau into the *ptl-1* mutant rescued the touch insensitivity, it did not significantly rescue the neuronal or life span deficits [67]. Curiously, human Tau expression in the wild-type *C. elegans* seemed to be toxic, as animals were touch-insensitive and had more neuronal defects with shorter life span [67]. These observations suggest that not all functions of Tau are conserved, and exogenous human Tau, even in its wild-type form, could be toxic either by dominant-negative or neomorphic effects. Like the case of APL-1, whether PTL-1 forms aggregates in aging *C. elegans* neurons is undetermined. These studies highlight the species-specific features of β-amyloid precursor, Tau, and probably other proteins implicated in human neurodegenerative diseases. Therefore, extreme care should be taken when interpreting data of expressing human disease-related proteins in the worm cells.

Interestingly, recent studies have shown that cellular proteins do show a tendency to form insoluble aggregates in old *C. elegans*, which could be suppressed by a life-extending *daf-2* mutation [72]. David et al. further demonstrated that such protein aggregates, while not affecting the animals’ locomotion on its own, aggravated paralysis induced by the expression of a pathogenic polyglutamine Yellow Fluorescent Protein (Q35-YFP) [72]. One of the possibilities is that aggregates of innocuous, physiological proteins facilitate the nucleation of pathogenic molecules and increase their cellular toxicity. Another possibility is that these aggregates recruit cellular proteins required for neuronal function, which then leads to compromised cellular physiology in the neuron. Although aggregates of Tau, α-synuclein or polyglutamine proteins are a widely recognized finding in human neurodegenerative diseases, whether aggregation of non-pathogenic proteins normally occurs during human aging remains poorly understood. Studies in *C. elegans* offer a good starting point to address this issue, which is central to the protein aggregation theory of cellular senescence.

## Conclusions

Even with several major differences in the architecture and molecular composition, *C. elegans* neurons display cardinal features of aging that are common to the nervous system in mammals. Moreover, the revelation that conserved pathways such as insulin signaling and JNK/MAPK pathways regulate *C. elegans* neuronal aging raises the possibility that these pathways also regulate human brain aging. However, studies in *C. elegans* also revealed significant variation in neuronal aging, even among neurons of the same type in the same individual animals. Are there any specific factors governing these seemingly “stochastic” aging episodes? What are the subcellular events that lead to a progressive dismantling of neuronal structures during normal aging? How does signaling between different tissues coordinate the speed of cellular senescence across the entire organism? Answers to each of these questions require careful examination of aging phenomena in individual animal models, before any ambition of translational aging research can be justified. The robust genetics and accessibility to live imaging make *C. elegans* a promising system to screen both for genes that regulate neuronal aging, and for chemicals that could potentially modify cellular aging processes. It is tantalizing to imagine that a set of common rules govern the demise of 302 neurons over two to three weeks in worms, and the decline of hundreds of billions of neurons over 20 years, in a *Homo sapiens*.

## Competing interests

The authors declared that they do not have financial or non-financial competing interests.

## Authors’ contributions

C-H Chen, Y-C Chen, H-C Jiang, C-K Chen and C-L Pan wrote the paper and prepared the Figures. All authors read and approved the final manuscript.
